# Social Media Application as a New Paradigm for Business Communication: The Role of COVID-19 Knowledge, Social Distancing, and Preventive Attitudes

**DOI:** 10.3389/fpsyg.2022.903082

**Published:** 2022-05-19

**Authors:** Songbo Yu, Jaffar Abbas, Anca Draghici, Oriana Helena Negulescu, Noor Ul Ain

**Affiliations:** ^1^Institute of Food and Strategic Reserves, Nanjing University of Finance and Economics, Nanjing, China; ^2^School of Media and Communication, Shanghai Jiao Tong University, Shanghai, China; ^3^Faculty of Management in Production and Transportation, Politehnica University of Timisoara, Timisoara, Romania; ^4^Faculty of Economic Sciences and Business Administration, Transilvania University of Brasov, Brasov, Romania; ^5^School of Management Sciences, Quad-i-Azam University, Islamabad, Pakistan

**Keywords:** business network, social media use, COVID-19, social distancing, COVID-19 knowledge

## Abstract

Business firms and the public have encountered massive consequences of the COVID-19 pandemic. This pandemic has become the most significant challenge and influenced all communities. This research study focuses on exploring the relationship between COVID-19 knowledge, social distancing, individuals' attitudes toward social media use, and practices of using social media amid the COVID-19 crisis. This study examines how attitudes toward social media use mediate the linkage between COVID-19 knowledge, social distancing, and practices for social media use. This survey uses a non-probability convenience sampling approach to collect samples and recruit willing respondents with their consent for data collection. This study recorded the feedback from 348 participants who encountered the indirect/direct effects of nationwide lockdowns, restrictions on social gatherings, and COVID-19 infection. The findings validate the proposed hypotheses for their direct effects and indicate significant β-values, *t*-statistics, and the *p*-values at *p* <0.001. The results validate a relationship between the COVID-19 knowledge of and social distancing practices. Similarly, the results approved a positive link between social distancing and attitudes toward social media use amid COVID-19. The findings validate the relation between social distancing and attitudes toward social media use during COVID-19 challenges (β*-*value = 0.22 and *t*-statistics = 3.078). The results show the linkage between attitudes toward social media use and practices of using social media (β-value = 0.41, and *t*-statistics = 7.175). Individuals' attitude toward social media use during COVID-19 mediates the connection between COVID-19 knowledge and COVID-19 practices of using social media use. The results validate the first mediation at β-value = 0.21 and *t*-statistic = 5.327. Similarly, the findings approve that attitudes toward social media use in the pandemic have positively mediated the relation between distancing and practices for social media use amid the crisis of COVID-19 (β-value = 0.09 and *t*-statistic = 2.633). The findings indicate how people have been indulged in social media to pave their business communication needs. The results provide valuable insights for the global business community. This study provides a systematic and holistic research model that helps in exploring the consequences of COVID-19. The generalizability of the findings provides valuable directions for future research related to the current pandemic.

## Introduction

The immense use of social media during the pandemic has been observed not only among individuals but also among business organizations as well (Haman, 2020; Shafi et al., 2020; Zhao and Zhou, 2021). Due to COVID-19, different businesses have faced many health and business operations problems (Abbas et al., 2021; Mubeen et al., 2021a; Aman et al., 2022; Ge et al., 2022; Liu et al., 2022; Rahmat et al., 2022). These challenges refer to the loss due to lost customers and supply chain disruptions (Moradi et al., 2021; Wang Y. et al., 2021; Zhou et al., 2021; Aqeel et al., 2022; Fu and Abbas, 2022; Mamirkulova et al., 2022). Firms have encountered problems, such as health problems, changes in foreign/export orders, and raw material shortages (Abbas, 2020b; Aman et al., 2021; Aqeel et al., 2021; Azadi et al., 2021; Khazaie et al., 2021; Lebni et al., 2021; Paulson et al., 2021; Shoib et al., 2021; Li et al., 2022). Vaccine availability is a challenging issue for firms to keep their employees healthy (Su et al., 2021a,b,c), and firms have faced disruptions in the transportation problems due to the global crisis (Shafi et al., 2020). However, these issues have been resolved under this pandemic considering the extensive use of social media platforms to approaching their customers (Abbas, 2020a; Mubeen et al., 2020, 2021b; Shuja et al., 2020; Azizi et al., 2021; Maqsood et al., 2021; NeJhaddadgar et al., 2022). Technological applications (Abbas et al., 2021; Lebni et al., 2021; Su et al., 2021c) and the supply demand curve are helpful for online delivery through e-commerce (Mason et al., 2021a,b). This pandemic has changed the string of knowledge, attitudes, and practices (KAP) toward social media use as a marketing tool and approaching new online markets (Mason et al., 2021a).

Apart from entertainment, social media use has proven effective during natural calamities such as floods, wars, earthquakes, riots, or lockdowns, probably because of its ease of usability, accessibility, and availability as a simple mode of communication (Rosenberg et al., 2018). In such situations, social platforms have proven to be integral in accessing real-time information about the happenings around the world and socially interacting with others, especially during the pandemic it remained essential (Jogezai et al., 2021). Social media use increased during the pandemic (Zhao and Zhou, 2021) owing to social distancing and quarantining of the individuals to prevent the spread of infection caused by COVID-19. Previous studies have been carried out on COVID-19 from different perspectives like digital learning during the emergence of COVID-19 virus (Hasan and Bao, 2020; Aditya, 2021; Chaturvedi et al., 2021; Deshpande and Mhatre, 2021; Smith et al., 2021), its impact on the economies of different countries (Hasan and Bao, 2020; Ye et al., 2020; Ali et al., 2021; Bhattacharya and Banerjee, 2021; Cuschieri and Grech, 2021; Delbiso et al., 2021; Donnarumma and Pezzulo, 2021; Klasche, 2021; Mahi et al., 2021; Prempeh, 2021; Roy et al., 2021), its role in the global health crisis (Abdalla et al., 2021; Ankrah et al., 2021; Chaturvedi et al., 2021; Chirisa et al., 2021; Donnarumma and Pezzulo, 2021; Hannam-Swain and Bailey, 2021; Klasche, 2021; Prempeh, 2021; Sarfraz et al., 2021; Wang et al., 2021; Zhao and Zhou, 2021) and the worst of all its impact on the mental wellbeing of people (Ciotti et al., 2020; Elmer et al., 2020; Filipova et al., 2020; Lee, 2020; Serafini et al., 2020; Adom et al., 2021; Chaturvedi et al., 2021; Coupet et al., 2021; Das and Bhattacharyya, 2021; Deshpande and Mhatre, 2021; Hannam-Swain and Bailey, 2021; Kareem, 2021; Li and Cao, 2021; Pandya and Lodha, 2021; Saha et al., 2021; Tonkin and Whitaker, 2021; Xiong et al., 2021). However, understanding the role of online social media use to meet the social needs after the closure of physical social interactive places amid COVID-19 is critical (Haman, 2020; Jogezai et al., 2021). The current study explores how the attitudes and practices of people in Pakistan regarding social media use amid COVID-19 get affected after getting knowledge about the severity of COVID-19.

COVID-19 infection and its transmission can be prevented by abiding by the proposed guiding principles following the World Health Organization (WHO) protocols, which are social distancing, hand washing, elbow sneezing, wearing masks, and quarantining COVID-19 suspects at the top of the list (World Health Organization, 2021). The use of social media sites like Facebook, Twitter, and Instagram satisfied the social needs of information, entertainment, and interpersonal communication of people who were staying distanced and quarantined due to the fear of spread. Not only this, quarantine for virus suspects has become easier due to social media use during these days of isolation. Although these escalations caused addiction in social media use behaviors of people (Purnama and Susanna, 2020), social media still have the ability for learning and electronic communication, and an adequate use of social media has not been studied profoundly in this pandemic.

Scholars have employed the KAP theory to understand the pandemic (Al-Hanawi et al., 2020; Andrade et al., 2020; Ferdous et al., 2020; Yousaf et al., 2020; Zhong et al., 2020; Alqahtani et al., 2021a,b; Kumar et al., 2021; Lee et al., 2021). KAP is a behavioral change theory that is commonly used in the literature to identify the knowledge gap, behavioral patterns, and predicting different behavioral changes among different socioeconomic groups for implementing effective health interventions (Lee et al., 2021). Despite the fact that COVID-19 has been widely studied through the lens of the KAP theory, the existing literature still has a significant gap regarding the integration of KAP toward social media use after the spread of COVID-19 with its severity and seriousness. Therefore, the main objective of this study is to understand how COVID-19 knowledge urges people to socially distance themselves and use social media (Aqeel et al., 2021; Yoosefi Lebni et al., 2021). How attitudes develop, which ultimately indulges people to experience social media use during this pandemic where social distancing and quarantining are the normal routine.

This proposed research model addresses the literature gap by probing the effects of preventive protocols, such as social distancing, COVID-19 knowledge, attitudes toward social media use amid COVID-19, and practices for social media use amid COVID-19. This research framework offers a persistent step in addressing the literature gap, and the study probes the effects of the selected variables on the practices of using social media amid COVID-19. The study addresses the gap and identifies limitations from the past literature on the relationship between COVID-19 knowledge and social distancing measures through the mediating role of attitudes toward social media use amid COVID-19. Accordingly, this research paper hypothesizes the logical reasoning to probe the association between COVID-19 knowledge, social distancing, and social media practices during the pandemic crisis.

Keeping in view the abovementioned debate, this research paper has formulated the following research questions:

1. How does COVID-19 knowledge correlate with attitudes toward social media use?
2. How does COVID-19 knowledge correlate with social distancing?
3. How social distancing has contributed to shaping attitudes toward social media use amid COVID-19?

## Theoretical Foundation

The KAP theory is a contemporary philosophy, which is extensively used to measure the KAP toward a certain situation (Zhong et al., 2020). It shows the integrated information on the understanding and awareness about certain concepts, how people perceived and then implemented it (Yousaf et al., 2020; Lee et al., 2021). The problems of awareness and developing an attitude among the population during COVID-19 are critical challenges, and some previous studies have discussed these problems. Previous KAP studies have examined in the form of surveys to assess the public about their level of understanding regarding knowledge on the particular social phenomenon, the attitudes, and their tendency to practice those guidelines. For example, Lee et al. (2021) have studied the KAP survey results in Korea through the COVID-19 time that how people were building up an attitude and integrating the knowledge in their practices to avoid the pandemic.

In a study, Kumar et al. (2021) had argued about the KAP addressing the guidelines among the students of Bangladesh. Similarly, in another study conducted in Bangladesh, Ferdous et al. (2020) have argued that the awareness in the general public and control measures could radically influence the KAP toward COVID-19. Alqahtani et al. (2021b) had also conducted a study in Saudi Arabia measuring the knowledge, attitude, and practical inclination of the general population about COVID-19 while one more study with similar parameters was conducted in Cape Verdean (Carvalho Alves et al., 2021). It was argued that there is an unequal burden of COVID-19 in socially deprived areas, ethnically marginalized groups, and poor people, which was augmented by non-communication to health service providers (Lee et al., 2021).

The current study has adopted the KAP theory for formulating the framework for this study. As Andrade et al. (2020) and Zhong et al. (2020) have argued that the population should follow the preventive measures given by local authorities to combat the pandemic and minimize its effect on the general public's health. For an effective introduction of COVID-19 about adequate prevention, people must be timely made aware of the fundamental principles regarding its severity, challenges, and the grave consequences it can offer (Andrade et al., 2020; Kumar et al., 2021; Rizwan et al., 2021). In this study, the use of KAP theory addresses that when the people are given due knowledge they intuitively develop a distancing attitude and get inclined toward social media use when they are limited to physical interaction with other people.

## Literature Review

COVID-19 disease is characterized by a contagious acute respiratory syndrome caused by the Corona Virus, which broke out in Wuhan, China in late 2019 after a few cases of pneumonia originated and it gradually swelled to the rest of the world (World Health Organization, 2021). In the pandemic situation, the intense use of social media by businesses has accessed new markets by reaching the potential customers through social marketing. A change in the social media behaviors of consumers has been seen recently since the COVID-19 pandemic (Mason et al., 2021b). Therefore, businesses have discovered new horizons in the capacity of small and medium enterprises to reach consumers on a mass level, which has been massively shaped through online social media marketing. This pandemic has given a new prospect to social media marketing to access new online markets adhering to supply chains, transportations, supply demand meet-up, and all issues faced in the pandemic.

This paper argues about the KAP of people in Pakistan regarding social media use amid COVID-19 and how they have used it to gain more business and approach new business markets by marketing their products through social media. Due to the global eruption of this viral disease throughout the world, the WHO declared it as an international emergency in January and 2 months later a pandemic as a result of the exponential spreading dynamics (World Health Organization, 2021). Regardless of the strict measures taken to prevent, avoid, and cure this viral pneumonia, it continued to affect thousands of people on a daily basis, all over the world. The fatality rate of COVID-19 remained higher at the global level. According to the WHO, around 276 million cases of COVID-19 have been reported. About 5.3 million people have died until 25 December 2021 from this severe pneumonic infection.

COVID-19 affected not only individuals facing the virus infection, but it also impacted the human society as a whole (Carvalho Alves et al., 2021). For this reason, as the COVID-19 spread started, the officials took several measures to avoid this spread. In an urge to keep the trajectory of COVID-19 flattened, educational institutes, including schools, colleges, and universities suspended all on-campus activities in the beginning; however, learning activities were converted to online classes and activities resumed (Kumar et al., 2021). Educational campaigns and awareness programs play a very vital role in waking up the consideration for taking measures to control diseases. Certain awareness and educational campaigns were initiated to let people know about the severity of the disease and how it could be prevented to get in touch with it. However, a very little proportion of the population adhered to the guidelines and measures taught to them. According to Zhong et al. (2020), the KAP toward social media use amid COVID-19 have been a major contributor to the general public's comprehension of the origin, transmission, and control of the disease and taking precautionary measures during this pandemic.

Local authorities used the social media platform to run their preventive measures and control campaigns as COVID-19 knowledge diverged them to interactive and communicative online social media rather than physical gatherings (Rizwan et al., 2021). So, during the pandemic, many cases of addictive social media use were reported where addiction to social media use is defined as an uncontrollable, a compelling, and an excessive use of social media (Andreassen, 2015). Knowledge and understanding about COVID-19 have intentionally forced individuals to distance themselves socially to avoid its spread, which induced the attitude toward social media among people. Elmer et al. (2020) and Rizwan et al. (2021) had argued that social crises like war, earthquakes, and pandemics affect the social orientations of individuals. The current pandemic of COVID-19 diverged the masses toward social media use for staying in touch, getting an education, or entertainment. In this attitude and inclination of indulging in using social media to compensate for their physical social interactions, an active social media use was observed (Baig and Waheed, 2016; Reuter et al., 2016; Azlan et al., 2020; Zhong et al., 2020; Carvalho Alves et al., 2021; Jogezai et al., 2021; Pandya and Lodha, 2021; Rizwan et al., 2021; Zhao and Zhou, 2021).

Due to the lockdown at the national level, sometimes smart lockdown, social distancing, and quarantining stopped lives resulting in developing the attitude of people in social media use that eventually turned into their addictions to social media use. In this critical phase of life, assessing the knowledge, perspectives, and practices regarding social media use during COVID-19 will have an important implication to take due precautionary measures to avoid its spread with physical social interactions. In this case, the owed knowledge, a reactive attitude, and vigorous practices regarding COVID-19, after its knowledge has created, the awareness to observe social distancing or quarantining, attitudes toward social media use amid COVID-19 rather than physical socialization, is still unexplored.

### Research Framework

This paper is the first research that aims to investigate the relationship between COVID-19 knowledge, social distancing, individuals' attitudes toward social media use in COVID-19, and practices for social media use amid the COVID-19 crisis. The study examines how individuals' attitudes toward social media use amid COVID-19 mediate the relationship between COVID-19 knowledge, social distancing, and practices for social media use amid the COVID-19 disaster. This study has chosen four variables to develop and present the research model. The study framework has taken COVID-19 knowledge and social distancing as independent variables (IVs), and practices for social media use amid COVID-19 are dependent variables (DVs). At the same time, the mediating variable is an attitude toward social media use amid COVID-19.

**Figure 1:**
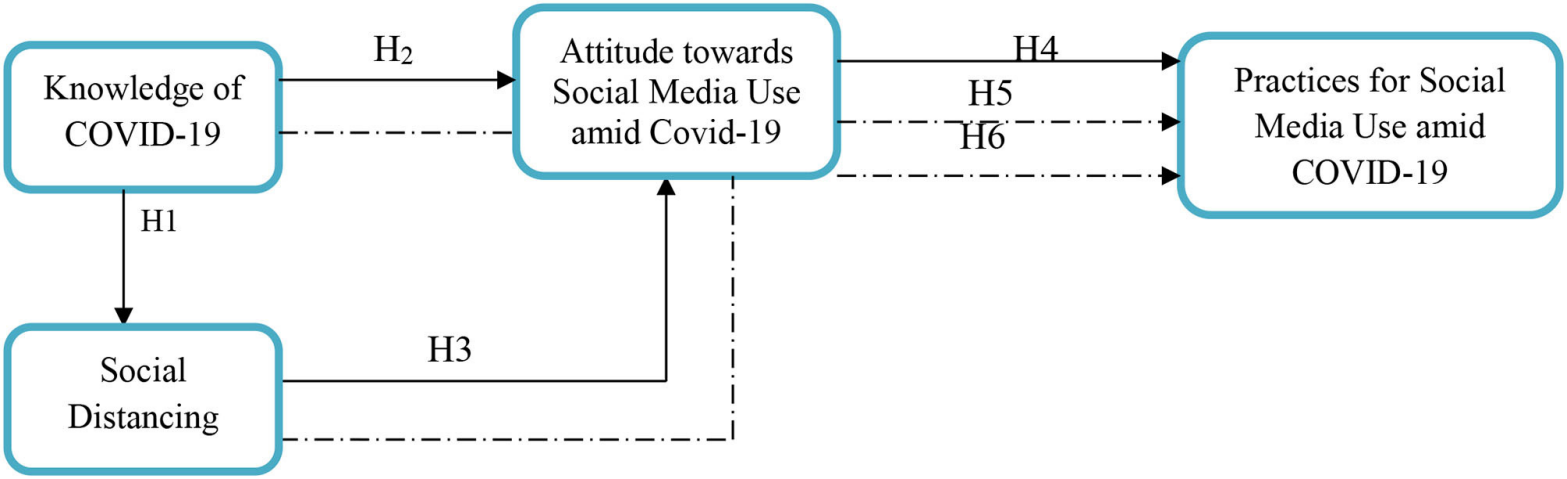
Theoretical framework. The proposed research framework shows selected variables; COVID-19 knowledge, social distancing, individuals' attitudes toward social media use in COVID-19, and practices for social media use amid the COVID-19 crisis.

The current study offers to fill the vacuum in the literature by evaluating the impact of COVID-19 knowledge on social distancing that consequently leads to the development of an attitude toward social media use amid the pandemic and nurtures the social media use behavior. The following framework gives a pictorial description of the theoretical grounds of this study. Figure 1 shows the study framework.

Based on the literature review and the devised theoretical framework, this study has formulated the following hypotheses:

H1: There is a positive relationship between COVID-19 knowledge and social distancing.
H2: There is a positive association between COVID-19 knowledge and attitude toward social media use amid COVID-19.
H3: There is a positive link between social distancing and attitude toward social media use amid COVID-19.
H4: There is a positive connection between attitudes toward COVID-19 containment and practices for social media use amid COVID-19.
H5: Attitude toward social media use amid COVID-19 mediates the relationship between COVID-19 knowledge containment and practices for social media use amid COVID-19.
H6: There is a positive mediation relationship between attitudes toward social media use amid COVID-19, social distancing, and practices for social media use during the pandemic.

The abovementioned framework of this study is based on the KAP theory of medical psychology, also known as behavior change theory. It proposes that the behavior change of the individuals is based on three phases, i.e., acquiring knowledge about a particular matter, generating an attitude toward that matter, and then forming individual behaviors.

## Materials and Methods

### Study Design

This analytical study explores empirical relationships using a descriptive observational, cross-sectional, deductive, and quantitative approach (Pitafi et al., 2018a,b; Farashah and Blomquist, 2019).

#### Study Location

The geographical location for this study is the country of Pakistan.

#### Participants

This study has considered respondents from different locations residing in Pakistan as the population study. The investigators collected desired sample data in 2021 (Pitafi et al., 2018a,b), when the pandemic had decreased a bit and people had recovered from the consequences of COVID-19. Therefore, the investigators have drawn a sample from the Pakistani population who suffered a pandemic infection. At the same time, people have also started to use online social media to communicate and interact with each other. This survey applied non-probability convenience sampling to collect data for further analysis (Avotra et al., 2021a). The investigators recruited participants who were available and willing to participate in the survey and obtained respondents' prior consent for data collection. This study received responses from 348 participants who faced COVID-19 infection.

#### Eligibility Criteria

This study set the eligibility criteria, and any male or female resident of Pakistan was eligible to participate in this study. Inclusion was based on respondents who mentally or physically faced the effects of COVID-19. The total number of questionnaires distributed to participants was 400. The researchers received 348 valid questionnaires for analysis. Respondents' response rate was 87%. This study excluded incomplete questionnaires and rejected them as they indicated insufficient and unanswered questionnaires.

#### Data Collection Procedure

Self-administered questionnaires were distributed by the investigators of this study to receive participants' feedback. This study used a self-administered scale to avoid vagueness in understanding the questions. Study participants were educated about the purpose of the survey and assured them of data confidentiality. Survey forms were distributed among recruited people residing in various locations in Pakistan. The researchers allowed participants to have a time of 4 weeks to return the filled forms. The survey forms were received and screened, and only useful feedback for data analysis was included (Kaur and Anand, 2018).

### Data Collection Instruments

This study collected the desired information through a 29-item questionnaire based on previous studies. The chosen questionnaire consists of two main parts (Abbas et al., 2019b; Farzadfar et al., 2022). The first part deals with the demographic profile of the respondents, and the second part consists of four further subsections, each subsection containing items dealing with each variable studied. The knowledge about COVID-19 has an IV. It consists of 11-items adapted from a previous study (Carvalho Alves et al., 2021). This scale measured the knowledge of people about COVID-19. The second and third parts contained the mediating variables of social distancing, and the study measured this with a four-item scale (Jogezai et al., 2021). This survey measured people's attitude toward social media use amid COVID-19 using a 10-item scale taken from the literature (Jogezai et al., 2021). Likewise, this study assessed DV, practices toward social media use amid COVID-19 with a four-item scale adopted from a past study (Jogezai et al., 2021). The study developed a questionnaire on a five-point Likert scale and indicated responses ranging from 1 to 5. Strong agreement was displayed with 5, while 1 indicated strong disagreement (where 1 = Strongly disagree, 2 = Disagree, 3 = Neutral, 4 = Agree, and 5 = Strongly agree). Table 1 shows demographic analysis of the participants.

**Table 1:** Demographic analysis.

**Demographics**	**Frequency**	**Percentage**
**Gender**		
Male	209	60.05%
Female	139	39.9%
**Age**		
15–20	103	29.59%
21–25	101	29.02%
26–30	72	20.68%
31 and above	72	20.68%
**Education**		
Bachelors	142	40.80%
Masters	115	33.04%
Ph.D. and others	91	26.14%
**Marital status**		
Single	167	47.98%
Married	108	31.03%
Divorced	42	12.06%
Widow	31	8.90%
**Nature of employment**		
Contract	78	22.41%
*Ad hoc*	102	29.31%
Permanent	64	18.39%
Unemployed	104	29.88%

*n = 348*.

### Statistical Analysis

In this study, the data have been analyzed with the help of statistical software Smart PLS version 3.3 for partial least-squares structural equation modeling (PLS-SEM) (Latif, K. et al., 2020). The demographic profile of the respondents was analyzed using the frequencies and percentages obtained for the different questions mentioned earlier (Aman et al., 2019; Pitafi et al., 2020a). These demographic profile questions included age, gender, marital status, education, and nature of employment of the respondents. The second part of the questionnaire was used for hypothesis testing (NeJhaddadgar et al., 2020; Pitafi et al., 2020b; Yao et al., 2022). The analysis of the data for hypotheses testing was based on two phases. The first is the measurement model estimation, and the second is the structural model estimation. In the first phase of measurement model estimation, the survey data were screened for valid and reliable items on the scale. The discriminant and convergent validities of the data were measured (Marchena-Giráldez et al., 2021). In addition, the tests used for the validity checking were factor loadings, average variance extracted (AVE), heterotrait-monotrait (HTMT) ratio, Fornell and Larcker criteria, along with *f*^2^ and *R*^2^ statistics. The hypotheses were tested for acceptance or rejection using the values, *t*-statistics, and *p*-values obtained for each hypothesis.

## Results

### Participants' Profile Analysis

Respondents' demographic profile obtained included 60% male and 40% female. The ages of the respondents were all roughly the same between the two age categories, such as 15–20 and 21–25, which accounts for 30% of the participants. About 21% of the respondents are from the other two categories. Among the education categories, bachelor's degree participants showed the highest frequency of around 40%, while 33% had master's degrees and 26% had PhD and other degrees/diplomas. The marital status of the respondents showed that the highest 48% were single and 31% were married. In addition, 12% of the respondents were divorced and 9% were widowed. Regarding the nature of employment, 29.31% of the respondents were working on an *ad hoc* basis and 29.88% were unemployed. Likewise, 22.5% were contractual employees. Permanent employees accounted for 30%.

### Descriptive Analysis

Table 2 shows the values of mean, standard deviation (SD), skewness, and excess kurtosis, addressing the descriptive statistics of the data.

**Table 2:** Mean, standard deviation (SD), excess kurtosis, and skewness analysis (*n* = 348).

**Variables**	**Items**	**Mean**	**SD**	**Excess kurtosis**	**Skewness**
Attitude toward social media	AttSM1	3.828	1.014	−0.378	−0.596
use amid COVID-19	AttSM2	3.675	1.029	0.132	−0.732
	AttSM3	3.583	1.065	−0.188	−0.628
	AttSM4	3.632	1.128	−0.532	−0.555
	AttSM5	3.764	1.051	−0.397	−0.635
	AttSM6	3.664	1.064	−0.125	−0.693
	AttSM7	3.773	1.013	0.183	−0.749
	AttSM8	3.98	1.143	0.475	−1.108
	AttSM9	3.764	1.049	−0.068	−0.718
	AttSM10	3.678	1.069	0.059	−0.746
Knowledge about COVID-19	KN1	4.006	1	1.481	−1.276
	KN2	3.911	1.08	0.66	−1.071
	KN3	3.908	0.999	0.545	−0.962
	KN4	4.049	1.127	1.048	−1.318
	KN5	3.816	1.048	−0.082	−0.77
	KN6	3.853	1.058	0.31	−0.918
	KN7	3.922	1.063	0.856	−1.144
	KN8	3.813	1.084	0.46	−0.996
	KN9	3.807	1.142	0.416	−1.023
	KN10	3.724	1.063	−0.096	−0.698
	KN11	3.911	1.227	0.255	−1.103
Social distancing	SD1	0.859	0.098	−0.69	3.773
	SD2	1.055	0.281	−0.88	3.802
	SD3	1.041	0.559	−0.972	3.813
	SD4	1.095	0.309	−0.94	3.845
Practices for social media	SMB1	1.035	0.908	−1.149	3.943
use amid COVID-19	SMB2	1.1	0.653	−1.067	3.856
	SMB3	1.047	0.617	−1.05	3.899
	SMB4	1.048	0.468	−0.966	3.882

Skewness threshold values mentioned in the literature is that the values should be between −3 and +3. Whereas, for kurtosis, the values should be between −10 and +10 to keep the data symmetrical and normally distributed. Structural equation modeling is a robust analytical method that does not show a significant deviation in the drawn results due to the violation of values (Griffin and Steinbrecher, 2013). However, the values in the present study display values that congest between −1.5 and +1.5, which confirms that data are typically normal and suitable for running the SEM analysis.

### Measurement Model Estimation

**Figure 2:**
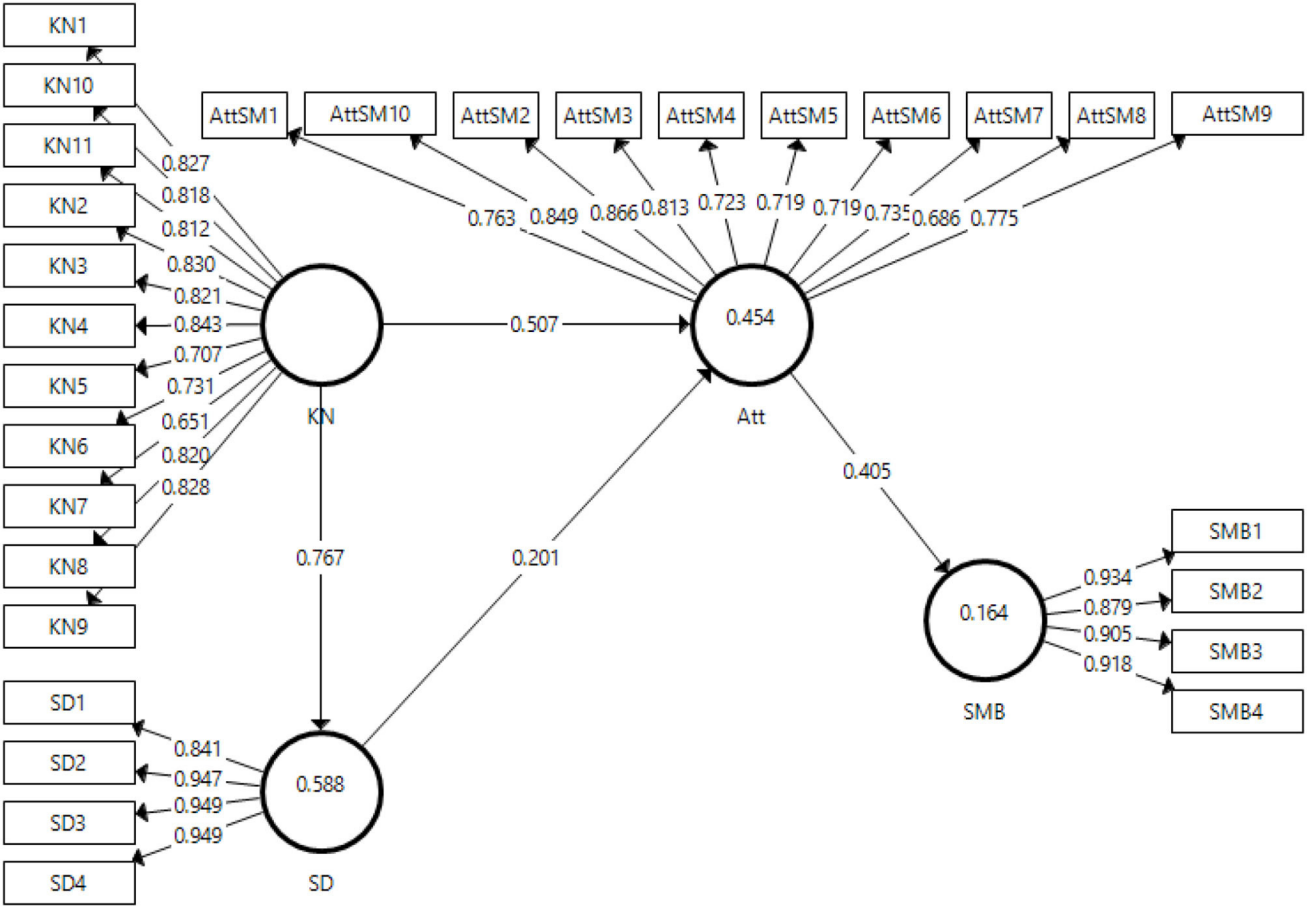
Measurement model algorithm.

This paper incorporated a statistical software Smart PLS version 3.3.3 to obtain the measurement model algorithm for the results, as indicated in Figure 2. This software helps generate preliminary validity and reliability results of the received data. It includes convergent validity, discriminant validity, reliabilities, factor loadings, *R*^2^, and *f*^2^ statistics. PLS-SEM is a statistical tool used by some researchers in their research studies. It uses robust, superior, flexible, and adequate statistical tools to generate sufficiently competent analytical models (Lotfi et al., 2018; Latif, K. F. et al., 2020; Avotra et al., 2021b).

#### Factor Loadings, Reliabilities, and AVE

The current study measured the convergent validity for the chosen variables and applied statistical tests for the factor loadings of the items, along with AVE, Cronbach-α reliabilities, and composite reliabilities. Table 3 shows the results obtained for these tests. The threshold value mentioned in the literature for Cronbach-α reliability and composite reliability is 0.7, while AVE is mentioned at 0.5 (Hair et al., 2017). All the values obtained in this study for reliabilities and AVE are above the threshold mentioned in the literature, along with the factor loadings. The cutoff value reported in the literature for factor loadings is 0.7 or 0.45 or above for small samples (Abbas et al., 2019a). However, the values obtained in this study were all well above the thresholds mentioned above. The lowest value of factor loadings in this study was 0.651 for the KN7 term. As the reliability and AVE of the overall variable were within acceptable limits, the study has not removed this item. Therefore, considering the results obtained, there is convergent validity of the variables in this study.

**Table 3:** It shows factor loadings, composite reliabilities, and average variance extracted (AVE) and convergent validity.

**Variables**	**Factor loadings**		**Cronbach alpha**	**Composite reliability**	**AVE**	**Convergent validity**
Attitude toward Social media	AttSM1	0.736	0.922	0.934	0.588	Yes
use amid COVID-19	AttSM2	0.849				
	AttSM3	0.808				
	AttSM4	0.728				
	AttSM5	0.736				
	AttSM6	0.746				
	AttSM7	0.745				
	AttSM8	0.694				
	AttSM9	0.775				
	AttSM10	0.849				
Knowledge about COVID-19	KN1	0.827	0.940	0.949	0.628	Yes
	KN10	0.818				
	KN11	0.813				
	KN2	0.830				
	KN3	0.821				
	KN4	0.843				
	KN5	0.707				
	KN6	0.731				
	KN7	0.651				
	KN8	0.820				
	KN9	0.828				
Social distancing	SD1	0.841	0.941	0.958	0.851	Yes
	SD2	0.947				
	SD3	0.949				
	SD4	0.949				
Practices for social media	SMB1	0.934	0.930	0.950	0.826	Yes
use amid COVID-19	SMB2	0.880				
	SMB3	0.904				
	SMB4	0.917				

*n = 348*.

Table 4 indicates the factor loadings and cross-loadings of the variables chosen in this model. The table shows that the items included in their corresponding variables show the highest loadings for the included factors. The remaining loadings shown on other variables are not sufficient to be included in these variables.

**Table 4:** Cross-loadings of the items based on the study variables.

**Influencing factors**	**Items**	**Att**	**KN**	**SD**	**SMB**
Attitude toward social media	AttSM1	**0.736**	0.341	0.345	0.269
use amid COVID-19	AttSM2	**0.849**	0.530	0.455	0.313
	AttSM3	**0.808**	0.436	0.393	0.303
	AttSM4	**0.728**	0.431	0.378	0.193
	AttSM5	**0.736**	0.524	0.589	0.384
	AttSM6	**0.746**	0.670	0.646	0.449
	AttSM7	**0.745**	0.557	0.402	0.250
	AttSM8	**0.694**	0.518	0.360	0.228
	AttSM9	**0.775**	0.358	0.315	0.266
	AttSM10	**0.849**	0.519	0.435	0.320
Knowledge about COVID-19	KN1	0.535	**0.827**	0.626	0.492
	KN10	0.558	**0.818**	0.551	0.431
	KN11	0.497	**0.813**	0.540	0.471
	KN2	0.594	**0.830**	0.612	0.414
	KN3	0.601	**0.821**	0.658	0.452
	KN4	0.594	**0.843**	0.652	0.455
	KN5	0.491	**0.707**	0.521	0.460
	KN6	0.555	**0.731**	0.513	0.405
	KN7	0.482	**0.651**	0.499	0.365
	KN8	0.493	**0.820**	0.594	0.499
	KN9	0.522	**0.828**	0.559	0.435
Social distancing	SD1	0.639	0.512	**0.841**	0.512
	SD2	0.543	0.597	**0.947**	0.493
	SD3	0.547	0.631	**0.949**	0.496
	SD4	0.526	0.678	**0.949**	0.470
Practices for social media	SMB1	0.410	0.568	0.544	**0.934**
use amid COVID-19	SMB2	0.365	0.496	0.453	**0.880**
	SMB3	0.325	0.477	0.479	**0.904**
	SMB4	0.392	0.491	0.468	**0.917**

*n = 348. The bold values indicate a decent score of the study items*.

#### Fornell and Larcker Criterion

Discriminant validities of the data were checked through the two most commonly used and strongly recommended tests of the Fornell and Larcker Criteria and the HTMT ratios (Franke and Sarstedt, 2019). The significance of the Fornell and Larcker test is that the top value in each column should be the highest of the rest of the column. The maximum values shown in Table 5 for the Fornell and Larcker test results were all significant in this study.

**Table 5:** Fornell and Larcker criteria.

	**Att**	**KN**	**SD**	**SMB**
Att	**0.767**			
KN	0.661	**0.792**		
SD	0.590	0.767	**0.922**	
SMB	0.405	0.561	0.536	**0.909**

*n = 348, Att, attitude toward social media use amid COVID-19; KN, knowledge about COVID-19; SD, social distancing; SMB, practices for social media use amid COVID-19. The bold values indicate a decent score of the study items*.

#### HTMT Ratio

Similarly, the most commonly used measure of discriminant validity is the HTMT ratio. The values obtained from the HTMT ratio are <0.85. The results are significant and do not show any multicollinearity, and the variables of the chosen model are distinct from each other. All the HTMT ratio values obtained in this study are <0.85, and 0.809 is the highest value in the analysis (Table 6). Thus, the acceptability of the data regarding discriminant validity has been shown (Franke and Sarstedt, 2019).

**Table 6:** Heterotrait-monotrait (HTMT) ratio.

	**Att**	**KN**	**SD**	**SMB**
Att				
KN	0.682			
SD	0.601	0.809		
SMB	0.416	0.598	0.570	

*n = 348, Att, attitude toward social media use amid COVID-19; KN, knowledge about COVID-19; SD, social distancing; SMB, practices for social media use amid COVID-19*.

#### R^2^ and f^2^ Statistics

*R*^2^-values obtained for the DV in this study show a decent outcome. The social distancing variable offers 58.5% of the highest *R*^2^-value. The results suggest that the knowledge of COVID-19 explains that the social distancing variable is influential. *R*^2^-values closer to 1 are considered more robust. Likewise, individuals' attitudes toward social media use amid COVID-19 present the entire model by 45.4%, while practices for social media use amid COVID-19 predict the proposed model by 16.4%, respectively (Mirayani et al., 2019). The values of *f*^2^ are usually small if they are <0.02. They will be moderate if below 0.15 and strong if the value is 0.35 or above. The highest value of *f*^2^ explains the relationship between COVID-19 knowledge and individuals' attitudes toward social media use amid COVID-19, which was 1.94. Similarly, the outcome between COVID-19 knowledge and social distancing is 1.42, which shows the most robust effect size. People's attitude toward practices for social media use amid COVID-19 showed a value of 1.96, which describes the strongest effect. Thus, all relationships identified in this study have shown significant effect sizes.

### Structural Model Estimation

**Figure 3:**
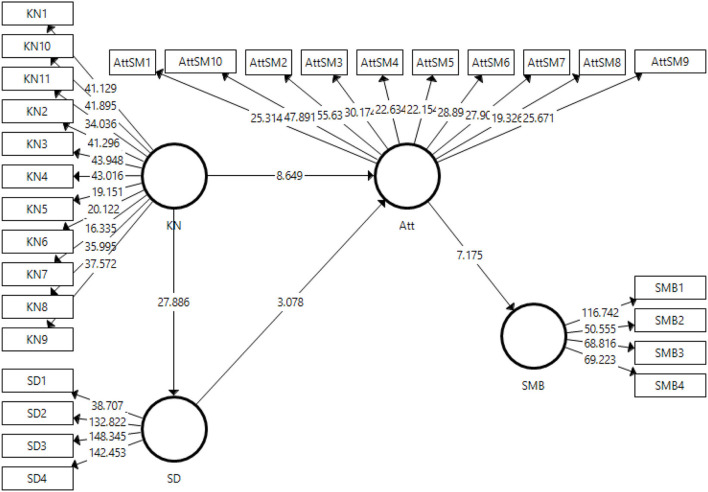
Structural model algorithm.

The next stage in the partial least squares structural model equation is the estimation of the structural model. Structural models estimate *t*-statistics, β*-*values, and *p*-values, which allow the study to accept or reject the hypotheses proposed. Figure 3 shows the structural model algorithm obtained for this study.

The obtained structural model shows the relationships of β-values. The standardized β-value explains the change in the DV with the unit change in IV. These are the measures of each relationship demonstrated in this study. Higher β-values show a significant effect on DV. On the other hand, the *t*-statistic represents and validates the magnitude of the β-value. In this study, the bootstrapping method has been used to evaluate and assess the significance of the proposed hypothesis.

#### Direct Effects

Table 7 presents the results of β-values, *t*-statistics, and *p*-values. The findings validate the four proposed hypotheses for the direct effects of the study at *p* < 0.001. Thus, the findings indicate significant *t*-statistics and substantial β-values. The first hypothesis, H1, states a positive connection between COVID-19 knowledge and social distancing. As indicated in Table 7, the results validate the association of COVID-19 knowledge and social distancing practices at a value of β = 0.76 and a *t*-statistic = 27.891. Similarly, the second hypothesis, H2, claims a positive association between COVID-19 knowledge and attitudes toward social media use amid COVID-19. The results approve H2 with a value of β = 0.51 and a *t*-statistic = 8.649. The third hypothesis describes a positive link between social distancing and attitude toward social media use amid COVID-19. The findings have approved the third hypothesis regarding the association of social distancing in developing attitudes toward social media use amid COVID-19 and accepted it at a value of β = 0.22 and a *t*-statistic = 3.078. The fourth hypothesis stated a positive affinity between attitude toward social media use and practices for social media use amid COVID-19. The results of this study validate the fourth hypothesis at a value of β = 0.41 and a *t*-statistic = 7.175.

**Table 7:** Hypothesis testing shows direct effects of the study variables.

**Paths**	**H**	**O**	**M**	**SD**	***T*-statistic**	***p***	**Results**
KN → SD	H_1_	0.766	0.766	0.027	27.891	0.000***	Accepted
KN → Att	H_2_	0.506	0.508	0.059	8.649	0.000**	Accepted
SD → Att	H_3_	0.203	0.205	0.066	3.078	0.000***	Accepted
Att → SMB	H_4_	0.406	0.412	0.057	7.175	0.000***	Accepted

*n = 348*,

***p < 0.001,

**p < 0.005,

*p < 0.05;

*H, hypothesis; O, original sample; M, sample mean; SD, standard deviation; Att, attitude toward social media use amid COVID-19; KN, knowledge about COVID-19; SD, social distancing; SMB, practices for social media use amid COVID-19*.

#### Indirect Effects

Table 8 indicates the results of the two indirect effects. This study formulated two hypotheses suggesting mediating effects.

**Table 8:** Indirect effects.

**Paths**	**H**	**O**	**M**	**SD**	***T*-statistic**	***p***	**Results**
KN → Atti → SMB	H_5_	0.205	0.209	0.039	5.327	0.000***	Accepted
SD → Att → SMB	H_6_	0.082	0.085	0.031	2.633	0.009**	Accepted

n = 348, ***p < 0.001, **p < 0.005, *p < 0.05; H, hypothesis; O, original sample; M, sample mean; SD, standard deviation; Att, attitude toward social media use amid COVID-19; KN, knowledge about COVID-19; SD, social distancing; SMB, practices for social media use amid COVID-19.

H5: Attitudes toward social media use amid COVID-19 mediates the relationship between the knowledge of COVID-19 containment and practices for social media use amid COVID-19. Attitudes toward social media use during COVID-19 is a mediating variable. The first mediation between COVID-19 knowledge and social media use practices in COVID-19 has been approved at a value of β = 0.21 and a *t*-statistic = 5.327.

H6: There is a positive mediation relationship between attitudes toward social media use amid COVID-19, social distancing, and practices for social media use during the pandemic. The results validate the second mediating variable, attitudes between social distancing and practices for social media use, at a value of β = 0.09 and a *t*-statistic = 2.633.

## Discussions

The present study has quantitatively measured the association of knowledge about COVID-19 with social distancing and the development of an attitude toward social media uses during COVID-19 and the consequent social media use behavior by business entities (Pitafi et al., 2018a,b; Anser et al., 2020; Rashid et al., 2020; Latif et al., 2021). It has further changed the avenue of reaching knowledge from a different perspective, attitude, and practices toward social media use as a tool of marketing and approaching new markets online (Mason et al., 2021a). In this current pandemic situation, the strong use of social media by businesses has enabled them to enter new markets by getting potential customers through social marketing (Pitafi et al., 2019; Rasheed et al., 2020; Wei et al., 2020). Due to different COVID-19 challenges, companies have faced many problems (Pitafi et al., 2018a,b; Kanwal et al., 2019; Latif, K. F. et al., 2020). These problems include the loss of existing customers, disruptions in supply chains and transportation, changes in foreign/export orders, and shortages of raw materials (Pitafi et al., 2020a,b; Shafi et al., 2020; Younis et al., 2020; Islam et al., 2021a,b,c; Pitafi and Ren, 2021). Therefore, companies at each level have found new horizons to reach the consumers at a mass level, which has been massively shaped through online social media marketing (Khan et al., 2020; Islm et al., 2021; Lai et al., 2021). COVID-19 has given a unique panorama to social media marketing to reach new online markets that adhere to supply chains, transportation, supply-demand meet-ups, and all problems faced in the pandemic (Khan et al., 2020; Islm et al., 2021; Lai et al., 2021).

The findings of this study show that COVID-19 has changed the mindset of people. It looks like the world is under the influence of the pandemic. The results obtained from this research model suggest a strong positive relationship between the knowledge of COVID-19 and social distancing, which validates H2 and fosters individuals' attitudes toward social media use. Table 7 indicates a positive link between COVID-19 knowledge and attitudes toward social media use amid COVID-19, which confirms H2. These findings are in accordance with previous studies conducted in this challenging scenario (Ferdous et al., 2020; Zhong et al., 2020; Lee et al., 2021). The study identifies that the practice of social distancing is positively associated with the development of attitudes toward social media use amid COVID-19. H3 claims the positive relationship between social distancing and attitude toward social media use amid COVID-19. The results have approved this hypothesized statement, as shown in Table 7. The results are consistent with recent literature. The scientific literature on the COVID-19 has also endorsed that when people are socially isolated and have no social interaction with other people, they start using social media as an alternate source of exchange of ideas.

The results are in line with previous studies (Ferdous et al., 2020; Alqahtani et al., 2021a,b; Rizwan et al., 2021). Eventually, people followed preventive protocols and started social media tools for communication purposes. Accordingly, H4 stated a positive and significant relation between individuals' attitudes toward COVID-19 social media use and practices for social media use amid COVID-19. The results validated it, as shown in Table 7. This study established that COVID-19 awareness and its consequences have affected public behavior. They understand the negligent effects of physical contact with the virus. People have distanced themselves socially to minimize the spread of the risks. As a result, people involved in social networks and started online interaction with others avoiding physical gatherings and dealings (H4). These findings are consistent with the past literature as studies related to the consequences of COVID-19 have reported that people involved in social media applications for various needs to avoid contact with the virus (Blackwell et al., 2017; Dutta, 2020; Chaturvedi et al., 2021; Jogezai et al., 2021; Rizwan et al., 2021; Zhao and Zhou, 2021).

This paper explores the H5 claim of how people's attitudes toward the use of social media apps mediate the relationship between COVID-19 knowledge, social distancing, and the actual practices of social media tools amid this pandemic crisis. The results (H5) are significant and validate this hypothesized statement, as shown in Table 8. This paper shows that when people know about the containment of the deadly virus, their attitude toward social media use in the pandemic makes them involve in the use of social media apps for their different life purposes. People can use these social media sources for their business activities, marketing, entertainment, or deepen their further knowledge about the current issues. The findings of this research have supported the hypothesis put forward. The findings support the results of the past literature in the same scenario from different regions worldwide (Jogezai et al., 2021; Rizwan et al., 2021; Zhao and Zhou, 2021). Similarly, H6 stated that attitudes toward social media use in the COVID-19 mediate the relationship between individuals' social distancing and practices for social media use during the pandemic. The study findings approve this claim, as indicated in Table 8. Social media applications and technological innovations have played a vital role to assist public and business organizations to meet their needs. This research has shown consistency with past research conducted on the consequences of the pandemic (Brem et al., 2021; Dahlke et al., 2021; Li et al., 2021; Mejía-Trejo, 2021; Ambrogio et al., 2022).

When people are knowledgeable and socially distanced, they tend to use social media to connect with others. They end up using them for everyday chores, so they depend on them for entertainment, education, and even shopping (Al-Rahmi et al., 2015; Karahanna et al., 2015; Blackwell et al., 2017; Dutta, 2020; Jogezai et al., 2021; Wilczewski et al., 2021; Zhao and Zhou, 2021). In contrast, some studies have found that social media use during COVID-19 is a source of stress (Ye et al., 2020; Rizwan et al., 2021). The adoption of social media use amid COVID-19 creates a network of acquaintances and friends on the internet. It leads them to develop an aptitude for its use. It makes users somewhat addicted to its use as they spend long hours online (Baig and Waheed, 2016; Zhao and Zhou, 2021). It builds more connections and relationships among users (Abbas et al., 2019a). This study entails that people aware of COVID-19 try to use social distancing and keep themselves in quarantine. Social media users try to save themselves from exposure to this life-taking disease and ultimately take refuge in social media. As social media use has become convenient for users in different ways, likewise, it includes studies, informal or formal social interaction, and work from home, entertainment, or shopping.

The present study has employed the structural equation modeling software based on Smart PLS version 3.3.3 for data analysis. This study analyzed the data in two phases. In the first stage, this study used measurement model estimation and performed preliminary data screening (Islam et al., 2021a,b,c; Wu et al., 2021). The proposed hypotheses were accepted or rejected based on structural model estimates. The discriminant and convergent validations were found to be acceptable for the study. Thus, the validity and the factor loading are confirmed *via* AVE, HTMT ratio, and the Fornell and Larcker criteria. Similarly, this study examined the reliability of the data *via* Cronbach-α reliability and composite reliability. The results indicated an α-value over 0.7, confirming that the data are reliable. The *R*^2^ represents the model fit, and its value was stronger and showed a substantial fit to the regression line.

This study shows that knowledge of COVID-19 has a positive and significant effect on social distancing between people. These survey findings are consistent with previous research (Besser et al., 2020; Das and Bhattacharyya, 2021; Jogezai et al., 2021; Kniffin et al., 2021). These studies show that, when people understand the severity of the pandemic and its life-threatening consequences, their defense mechanisms keep them away from others to avoid unknowingly contracting the virus. Likewise, current research finds that those aware of COVID-19 know the results of an incidental exposure to the virus. People follow social distancing measures and engage in online interactions with others. They avoid physical gatherings and transactions. These findings follow previous research in similar settings that also found that the spread of COVID-19 has engaged people in social media use and increased their reliance on social media (Blackwell et al., 2017; Dutta, 2020; Chaturvedi et al., 2021; Jogezai et al., 2021; Rizwan et al., 2021; Zhao and Zhou, 2021).

## Theoretical Contributions and Practical Implications

The findings of this study alarm the critical nature of being aware of the potentially hazardous threats of the COVID-19 virus that can take the lives of individuals. However, the proper use of social media for businesses has opened up new horizons, especially for small and medium enterprises to flourish by using social media marketing techniques to penetrate existing markets that were not physically accessible. It also helped organizations enter e-commerce to reach tiny chunks of potential consumers that would help them flourish and allow them to introduce new product lines or related products. The study also offers small businesses to make more composite moves to resolve the issues of supply chains, transportation, customer loss, and meeting demands by finding new customers to compensate. Controlling and adhering to the guidelines proposed by local authorities are essential to avoid contact with this virus, which in itself is a challenge in this pandemic time. The study's findings also encourage entrepreneurs to establish their business communications through social media among business communities and target markets. Apart from that, business already in operation should invest in their online training programs and avail the work from home opportunities to give employees liberty to enjoy flexible hours in their jobs.

Governing bodies should emphasize implementing preventive measures and hygiene protocols to avoid the transmission of this disease. Moreover, such an emphasis on preventive and control measures will contribute to the society at the mass level by diverging their mode of communication from physical to online, thus fulfilling the needs of the people without getting involved in physical dealings with each other. The results of the present study add to the body of literature related to social media use and KAP theory by identifying knowledge about natural calamities, developing an attitude toward the alternative way of communication, and finally getting involved in the use of online social media. To the best of our knowledge, this study is among the initial studies that have measured the aptitude of COVID-19 victims who indulge in social media use to run their personal and professional lives through the KAP theory. The present study offers a holistic and integrated research model that helps to investigate the consequences of COVID-19 by incorporating the KAP theory.

## Limitations and Recommendations

Despite the rigor of the study, some limitations need to be addressed in future studies. First of all, this is a quantitative cross-sectional study. Future studies can conduct longitudinal studies to distinguish trends in the four different waves of COVID-19 and consider live interviews and incorporate the mixed methods approach. Secondly, the investigators conducted this study in Pakistan, a developing country. The intensity of the COVID-19 virus had not been much severe as in European countries. Hence, future studies can show better and improved results by replicating this framework in Europe or other regions. Third, the present study has taken only one variable of social distancing. In future studies, more variables can be integrated into the KAP theory regarding COVID-19, considering tourism, intention to seek health services, and online learning outcomes. Additionally, future studies can investigate moderation effects.

## Conclusion

Health professionals have considered social distancing as the most effective preventive protocol to minimize the transmission of deadly COVID-19 virus to public and business communities. This study explores how the knowledge about the COVID-19 pandemic educates business communities and other individuals to follow social distancing protocols. Preventive protocols develop attitudes among people of social distancing. Since the outbreak of the pandemic (COVID-19), governing body authorities have implemented specific preventive strategies to take precautionary measures to control its exponential spread. This research study primarily focused on exploring the association between COVID-19 knowledge, social distancing, people's attitudes toward social media use in COVID-19, and practices for social media use amid the COVID-19 crisis. It also explored how attitudes toward social media use in COVID-19 have mediated the connection between COVID-19 knowledge, social distancing, and practices for social media use. This study has chosen four variables to develop and present this research model. All proposed hypotheses were tested and verified. COVID-19 knowledge leads people to indulge in social distancing. When people are quarantined or socially distanced, an attitude toward social media use mechanically develops.

This present study has explored how an attitude toward social media use develops due to COVID-19 knowledge and leads to the indulgence of people who use social media. In the current study, smart PLS structural equation modeling has been used to measure the relationship of social distancing on social media use to integrate the KAP theory. Variance-based structural equations of the model showed significant results for the hypotheses developed. Precisely, COVID-19 knowledge has been found to have a positive and significant effect on social distancing and the development of an attitude toward social media use amid COVID-19. Further, this attitude has been established as an essential precedent for people to follow social media use practices. Additionally, attitudes toward social media users have been a significant mediator among the relationships between social distancing and social media use during COVID-19, supporting the KAP theory of behavior change based on social media use amid the pandemic around the world.

## Data Availability Statement

The original contributions presented in the study are included in the article/supplementary material, further inquiries can be directed to the corresponding author/s.

## Author Contributions

JA and NA have conceptualized the idea, contributed to the framework of this study, project administration, supervision, idea conceptualization, writing the abstract, introduction, literature, discussion, and implications. SY, AD, ON, and NA have contributed to literature, methods, and discussion sections. SY, AD, ON, and JA contributed to the study expenses, revised, and critically reviewed the article. JA and AD have critically reviewed the revised manuscript. All authors made significant changes introduced at the proofing stage, contributed to the paper, edited, approved the final revised manuscript before submission, and approved the submitted version for publication.

## Conflict of Interest

The authors declare that the research was conducted in the absence of any commercial or financial relationships that could be construed as a potential conflict of interest.

## Publisher's Note

All claims expressed in this article are solely those of the authors and do not necessarily represent those of their affiliated organizations, or those of the publisher, the editors and the reviewers. Any product that may be evaluated in this article, or claim that may be made by its manufacturer, is not guaranteed or endorsed by the publisher.

## References

[B1] Abbas J.. (2020a). The impact of coronavirus (SARS-CoV2) epidemic on individuals mental health: the protective measures of pakistan in managing and sustaining transmissible disease. Psychiatr. Danub. 32, 472–477. 10.24869/psyd.2020.472 33370755

[B2] Abbas J.. (2020b). The role of interventions to manage and reduce COVID-19 mortality rate of the COVID-19 patients worldwide. Found. Univ. J. Psychol. 4, 33–36. 10.33897/fujp.v4i2.158

[B3] Abbas J. Aman J. Nurunnabi M. Bano S. (2019b). The Impact of Social Media on Learning Behavior for Sustainable Education: Evidence of Students from Selected Universities in Pakistan. Sustainability. 11:1683. 10.3390/su11061683

[B4] Abbas J. Mahmood S. Ali H. Raza M. A. Ali G. Aman J.. (2019a). The effects of corporate social responsibility practices and environmental factors through a moderating role of social media marketing on sustainable performance of business firms. Sustainability 11:3434. 10.3390/SU11123434

[B5] Abbas J. Wang D. Su Z. Ziapour A. (2021). The role of social media in the advent of covid-19 pandemic: crisis management, mental health challenges and implications. Risk Manag. Healthc. Policy 14, 1917–1932. 10.2147/RMHP.S284313 34012304 PMC8126999

[B6] Abdalla M. J. Said H. Ali L. Ali F. Chen X. (2021). COVID-19 and unpaid leave: Impacts of psychological contract breach on organizational distrust and turnover intention: mediating role of emotional exhaustion. Tourism Manag. Perspect. 39, 100854. 10.1016/j.tmp.2021.100854 34513584 PMC8419792

[B7] Aditya D. S.. (2021). Embarking digital learning due to COVID-19: are teachers ready? JOTSE 11, 104–116. 10.3926/jotse.1109

[B8] Adom D. Mensah J. A. Osei M. (2021). The psychological distress and mental health disorders from COVID-19 stigmatization in Ghana. Soc. Sci. Human. Open 4, 100186. 10.1016/j.ssaho.2021.100186 34250461 PMC8257423

[B9] Al-Hanawi M. K. Angawi K. Alshareef N. Qattan A. M. N. Helmy H. Z. Abudawood Y.. (2020). Knowledge, attitude and practice toward COVID-19 among the public in the kingdom of Saudi Arabia: a cross-sectional study. Front. Public Health 8, 217. 10.3389/fpubh.2020.00217 32574300 PMC7266869

[B10] Ali M. Gasca V. Schrier R. Pensa M. Brockman A. Olson D. P.. (2021). Social determinants and COVID-19 in a community health center cohort. J. Immigr. Minor. Health 24, 10–17. 10.1007/s10903-021-01320-6 34850318 PMC8631554

[B11] Alqahtani A. H. Alqahtani S. A. Alhodaib A. S. Al-Wathinani A. M. Daoulah A. Alhamid S.. (2021a). Knowledge, attitude, and practice (Kap) toward the novel coronavirus (covid-19) pandemic in a saudi population-based survey. Int. J. Environ. Res. Public Health 18, 5286. 10.3390/ijerph18105286 34065670 PMC8156526

[B12] Alqahtani A. H. Alqahtani S. A. Alhodaib A. S. Al-Wathinani A. M. Daoulah A. Alhamid S.. (2021b). Knowledge, attitude, and practice (KAP) toward the Novel Coronavirus (COVID-19) pandemic in a Saudi Population-based survey. Int. J. Environ. Res. Public Health 18, 5286. 10.3390/IJERPH18105286 34065670 PMC8156526

[B13] Al-Rahmi W. M. Othman M. S. Yusof L. M. Musa M. A. (2015). Using social media as a tool for improving academic performance through collaborative learning in Malaysian higher education. Rev. Eur. Stud. 7, 265–275. 10.5539/res.v7n3p265

[B14] Aman J. Abbas J. Lela U. Shi G. (2021). Religious affiliation, daily spirituals, and private religious factors promote marital commitment among married couples: does religiosity help people amid the COVID-19 crisis? Front. Psychol. 12, 657400. 10.3389/fpsyg.2021.657400 34421712 PMC8377757

[B15] Aman J. Abbas J. Mahmood S. Nurunnabi M. Bano S. (2019). The Influence of Islamic Religiosity on the Perceived Socio-Cultural Impact of Sustainable Tourism Development in Pakistan: A Structural Equation Modeling Approach. Sustainability. 11:3039. 10.3390/su11113039

[B16] Aman J. Abbas J. Shi G. Ain N. U. Gu L. (2022). Community wellbeing under china-pakistan economic corridor: role of social, economic, cultural, and educational factors in improving residents' quality of life. Front. Psychol. 12, 816592. 10.3389/fpsyg.2021.816592 35422725 PMC9004670

[B17] Ambrogio G. Filice L. Longo F. Padovano A. (2022). Workforce and supply chain disruption as a digital and technological innovation opportunity for resilient manufacturing systems in the COVID-19 pandemic. Comput. Ind. Eng. 169:108158. 10.1016/j.cie.2022.108158 35431410 PMC8993411

[B18] Andrade C. Menon V. Ameen S. Kumar Praharaj S. (2020). Designing and conducting knowledge, attitude, and practice surveys in psychiatry: practical guidance. Indian J. Psychol. Med. 42, 478–481. 10.1177/0253717620946111 33414597 PMC7750837

[B19] Andreassen C. S.. (2015). Online social network site addiction: a comprehensive review. Curr. Addict. Rep. 2, 175–184. 10.1007/S40429-015-0056-9/TABLES/1

[B20] Ankrah D. A. Agyei-Holmes A. Boakye A. A. (2021). Ghana's rice value chain resilience in the context of COVID-19. Social Sci. Human. Open 4, 100210. 10.1016/j.ssaho.2021.100210 34604735 PMC8469374

[B21] Anser M. K. Zaigham G. H. K. Imran Rasheed M. Pitafi A. H. Iqbal J. Luqman A. (2020). Social media usage and individuals' intentions toward adopting Bitcoin: the role of the theory of planned behavior and perceived risk. Int. J. Commun. Syst. 33, e4590. 10.1002/dac.4590

[B22] Aqeel M. Abbas J. Shuja K. H. Rehna T. Ziapour A. Yousaf I.. (2021). The influence of illness perception, anxiety and depression disorders on students mental health during COVID-19 outbreak in Pakistan: a web-based cross-sectional survey. Int. J. Human Rights Healthcare 15, 17–30. 10.1108/ijhrh-10-2020-0095

[B23] Aqeel M. Rehna T. Shuja K. H. Abbas J. (2022). Comparison of students' mental wellbeing, anxiety, depression, and quality of life during covid-19's full and partial (smart) lockdowns: A follow-up study at a 5-month interval. Front Psychiatry. 13:835585. 10.3389/fpsyt.2022.835585 35530024 PMC9067378

[B24] Avotra A. A. R. N. Chengang Y. Sandra Marcelline T. R. Asad A. Yingfei Y. (2021a). Examining the impact of E-government on corporate social responsibility performance: the mediating effect of mandatory corporate social responsibility policy, corruption, and information and communication technologies development during the COVID era. Front. Psychol. 12, 4221. 10.3389/fpsyg.2021.737100 34712183 PMC8545817

[B25] Avotra A. A. R. N. Chenyun Y. Yongmin W. Lijuan Z. Nawaz A. (2021b). Conceptualizing the state of the art of corporate social responsibility (csr) in green construction and its nexus to sustainable development. Front. Environ. Sci. 9, 541. 10.3389/fenvs.2021.774822

[B26] Azadi N. A. Ziapour A. Lebni J. Y. Irandoost S. F. Abbas J. Chaboksavar F. (2021). The effect of education based on health belief model on promoting preventive behaviors of hypertensive disease in staff of the Iran University of Medical Sciences. Arch. Public Health 79, 69. 10.1186/s13690-021-00594-4 33952339 PMC8097917

[B27] Azizi M. R. Atlasi R. Ziapour A. Abbas J. Naemi R. (2021). Innovative human resource management strategies during the COVID-19 pandemic: a systematic narrative review approach. Heliyon 7, e07233. 10.1016/j.heliyon.2021.e07233 34124399 PMC8183111

[B28] Azlan A. A. Hamzah M. R. Sern T. J. Ayub S. H. Mohamad E. (2020). Public knowledge, attitudes and practices towards COVID-19: a cross-sectional study in Malaysia. PLoS ONE 15, e0233668. 10.1371/JOURNAL.PONE.0233668 32437434 PMC7241824

[B29] Baig N. U. A. Waheed A. (2016). Significance of factors influencing online knowledge sharing: a study of higher education in Pakistan. Pakistan J. Comm. Social Sci. 10, 1–26. Available online at: https://www.proquest.com/docview/1833035541?pq-origsite=gscholar&fromopenview=true

[B30] Besser A. Flett G. L. Nepon T. Zeigler-Hill V. (2020). Personality, cognition, and adaptability to the COVID-19 pandemic: associations with loneliness, distress, and positive and negative mood states. Int. J. Ment. Health Addict. 2020, 1–20. 10.1007/s11469-020-00421-x 33230393 PMC7673240

[B31] Bhattacharya M. Banerjee P. (2021). COVID-19: Indices of economic and health vulnerability for the Indian states. Social Sci. Human. Open 4, 100157. 10.1016/j.ssaho.2021.100157

[B32] Blackwell D. Leaman C. Tramposch R. Osborne C. Liss M. (2017). Extraversion, neuroticism, attachment style and fear of missing out as predictors of social media use and addiction. Pers. Individ. Dif. 116, 69–72. 10.1016/j.paid.2017.04.039

[B33] Brem A. Viardot E. Nylund P. A. (2021). Implications of the coronavirus (COVID-19) outbreak for innovation: Which technologies will improve our lives? Technol. Forecast. Soc. Chang. 163:120451. 10.1016/j.techfore.2020.120451 33191956 PMC7648540

[B34] Carvalho Alves M. de F. Lima Mendonça M. da L. Xavier Soares J. de J. Leal S. D. V. dos Santos M.. (2021). Knowledge, attitudes and practices towards COVID-19: A cross-sectional study in the resident cape-verdean population. Social Sci. Human. Open 4, 100184. 10.1016/J.SSAHO.2021.100184 34308335 PMC8270755

[B35] Chaturvedi K. Vishwakarma D. K. Singh N. (2021). COVID-19 and its impact on education, social life and mental health of students: a survey. Children Youth Serv. Rev. 121, 105866. 10.1016/j.childyouth.2020.105866 33390636 PMC7762625

[B36] Chirisa I. Mavhima B. Nyevera T. Chigudu A. Makochekanwa A. Matai J.. (2021). The impact and implications of COVID-19: reflections on the Zimbabwean society. Social Sci. Human. Open 4, 100183. 10.1016/j.ssaho.2021.100183 34746754 PMC8558728

[B37] Ciotti M. Ciccozzi M. Terrinoni A. Jiang W. C. Wang C. Bin Bernardini S. (2020). The COVID-19 pandemic. Crit. Rev. Clin. Lab. Sci. 2020, 365–388. 10.1080/10408363.2020.1783198 32645276

[B38] Coupet S. Nicolas G. Louder C. N. Meyer M. (2021). When public health messages become stressful: managing chronic disease during COVID-19. Soc. Sci. Human. Open 4, 100150. 10.1016/j.ssaho.2021.100150 33880443 PMC8030736

[B39] Cuschieri S. Grech V. (2021). Protecting our vulnerable in the midst of the COVID-19 pandemic: lessons learnt from Malta. Public Health 198, 270–272. 10.1016/j.puhe.2021.07.043 34492507 PMC8354801

[B40] Dahlke J. Bogner K. Becker M. Schlaile M. P. Pyka A. Ebersberger B. (2021). Crisis-driven innovation and fundamental human needs: A typological framework of rapid-response COVID-19 innovations. Technol. Forecast. Soc. Chang. 169:120799. 10.1016/j.techfore.2021.120799 PMC9755532 36540548

[B41] Das M. Bhattacharyya A. (2021). Social distanciation through COVID-19: a narrative analysis of Indian Peri-Urban Elderly. Social Sci Human. Open 4, 100139. 10.1016/j.ssaho.2021.100139 34927054 PMC8665153

[B42] Delbiso T. D. Kotecho M. G. Asfaw F. M. (2021). Effects of COVID-19 imposed school closure on school feeding program in Addis Ababa, Ethiopia. Social Sciences and Humanities Open 4, 100185. 10.1016/j.ssaho.2021.100185 34927061 PMC8665152

[B43] Deshpande D. D. Mhatre C. K. (2021). A study of impact of online education on mental health and academic performance of children of project affected people studying at undergraduate level in Navi Mumbai. Rev. Gestão Inov. Tecnol. 11, 3866–3875. 10.47059/revistageintec.v11i4.2412

[B44] Donnarumma F. Pezzulo G. (2021). Moral decisions in the age of COVID-19: your choices really matter. Soc. Sci. Human. Open 4, 100149. 10.1016/j.ssaho.2021.100149 34927057 PMC8665354

[B45] Dutta D. A.. (2020). Impact of digital social media on indian higher education: alternative approaches of online learning during COVID-19 pandemic crisis. Int. J. Sci. Res. Publ. (IJSRP) 10, 604–611. 10.29322/ijsrp.10.05.2020.p10169

[B46] Elmer T. Mepham K. Stadtfeld C. (2020). Students under lockdown: comparisons of students' social networks and mental health before and during the COVID-19 crisis in Switzerland. PLoS ONE 15, 236337. 10.1371/journal.pone.0236337 32702065 PMC7377438

[B47] Farashah A. D. Blomquist T. (2019). Exploring employer attitude towards migrant workers: evidence from managers across Europe. Evid-Based HRM 8, 18–37. 10.1108/EBHRM-04-2019-0040

[B48] Farzadfar F. Naghavi M. Sepanlou S. G. Saeedi Moghaddam S. Dangel W. J. Davis Weaver N.. (2022). Health system performance in Iran: a systematic analysis for the Global Burden of Disease Study 2019. The Lancet. 399:1625–1645. 10.1016/s0140-6736(21)02751-3 35397236 PMC9023870

[B49] Ferdous M. Z. Islam M. S. Sikder M. T. Mosaddek A. S. M. Zegarra-Valdivia J. A. Gozal D. (2020). Knowledge, attitude, and practice regarding COVID-19 outbreak in Bangladesh: an online-based cross-sectional study. PLoS ONE 15, e0239254. 10.1371/JOURNAL.PONE.0239254 33035219 PMC7546509

[B50] Filipova T. Kopsieker L. Gerritsen E. Bodin E. Brzezinski B. Ramirez-Rubio O. (2020). Mental Health and the Environment: How European Policies Can Better Reflect the Impact of Environmental Degradation On People's Mental Health and Well-Being (Issue December). Available online at: https://ieep.eu/uploads/articles/attachments/2bfb2051-b305-4338-9770-ae8071320b1a/Mentalhealthandthe~environment.pdf?v=63775265428 (accessed January 15, 2022).

[B51] Franke G. Sarstedt M. (2019). Heuristics versus statistics in discriminant validity testing: a comparison of four procedures. Internet Res. 29, 430–447. 10.1108/IntR-12-2017-0515

[B52] Fu Q. Abbas J. (2022). Reset the industry redux through corporate social responsibility: the COVID-19 tourism impact on hospitality firms through business model innovation. Front. Psychol. 12, 795345. 10.3389/fpsyg.2021.795345

[B53] Ge T. Abbas J. Ullah R. Abbas A. Sadiq I. Zhang R. (2022). Women's Entrepreneurial contribution to family income: innovative technologies promote females' entrepreneurship amid COVID-19 crisis. Front. Psychol. 13, 828040. 35422737 10.3389/fpsyg.2022.828040 PMC9004668

[B54] Griffin M. M. Steinbrecher T. D. (2013). Large-scale datasets in special education research. Int. Rev. Res. Dev. Disabil. 45, 155–183. 10.1016/B978-0-12-407760-7.00004-9

[B55] Hair J. F. J. Hult G. T. M. Ringle C. M. Sarstedt M. (2017). A Primer on Partial Least Squares Structural Equation Modeling (PLS-SEM). London: Sage.

[B56] Haman M.. (2020). The use of Twitter by state leaders and its impact on the public during the COVID-19 pandemic. Heliyon 6, e05540. 10.1016/J.HELIYON.2020.E05540 33294685 PMC7695954

[B57] Hannam-Swain S. Bailey C. (2021). Considering Covid-19: Autoethnographic reflections on working practices in a time of crisis by two disabled UK academics. Soc. Sci. Human. Open 4, 100145. 10.1016/j.ssaho.2021.100145 34173514 PMC7997622

[B58] Hasan N. Bao Y. (2020). Impact of “e-Learning crack-up” perception on psychological distress among college students during COVID-19 pandemic: a mediating role of “fear of academic year loss.” Children Youth Serv. Rev. 118, 105355. 10.1016/j.childyouth.2020.105355 32834276 PMC7422835

[B59] Islam T. Islam R. Pitafi A. H. Xiaobei L. Rehmani M. Irfan M.. (2021a). The impact of corporate social responsibility on customer loyalty: the mediating role of corporate reputation, customer satisfaction, and trust. Sustain. Prod. Consump. 25, 123–135. 10.1016/j.spc.2020.07.019

[B60] Islam T. Pitafi A. H. Akhtar N. Xiaobei L. (2021b). Determinants of purchase luxury counterfeit products in social commerce: the mediating role of compulsive internet use. J. Retail. Consum. Serv. 62, 102596. 10.1016/j.jretconser.2021.102596

[B61] Islam T. Pitafi A. H. Arya V. Wang Y. Akhtar N. Mubarik S.. (2021c). Panic buying in the COVID-19 pandemic: a multi-country examination. J. Retail. Consumer Serv. 59, 102357. 10.1016/j.jretconser.2020.102357

[B62] Islm T. Meng H. Pitafi A. H. Ullah Zafar A. Sheikh Z. Shujaat Mubarik M.. (2021). Why DO citizens engage in government social media accounts during COVID-19 pandemic? A comparative study. Telem. Inform. 62, 101619. 10.1016/j.tele.2021.101619 PMC9758419 36568845

[B63] Jogezai N. A. Baloch F. A. Jaffar M. Shah T. Khilji G. K. Bashir S. (2021). Teachers' attitudes towards social media (SM) use in online learning amid the COVID-19 pandemic: the effects of SM use by teachers and religious scholars during physical distancing. Heliyon 7, e06781. 10.1016/j.heliyon.2021.e06781 33948511 PMC8080042

[B64] Kanwal S. Pitafi A. H. Pitafi A. Nadeem M. A. Younis A. Chong R. (2019). China–Pakistan Economic Corridor (CPEC) development projects and entrepreneurial potential of locals. J. Public Affairs 19, e1954. 10.1002/pa.1954

[B65] Karahanna E. Xin Xu S. Zhang N. (2015). Psychological ownership motivation and use of social media. J. Market. Theory Pract. 23, 185–207. 10.1080/10696679.2015.1002336

[B66] Kareem B.. (2021). Do global pandemics disrupt or seed transformations in cities? A systematic review of evidence. Soc. Sci. Human. Open 4, 100138. 10.1016/j.ssaho.2021.100138

[B67] Kaur H. Anand S. (2018). Segmenting Generation Y using the Big Five personality traits: understanding differences in fashion consciousness, status consumption and materialism. Young Consum. 19, 382–401. 10.1108/YC-03-2018-00788

[B68] Khan N. A. Khan A. N. Moin M. F. Pitafi A. H. (2020). A trail of chaos: How psychopathic leadership influence employee satisfaction and turnover intention via self-efficacy in tourism enterprises. J. Leis. Res. 52, 347–369. 10.1080/00222216.2020.1785359

[B69] Khazaie H. Lebni J. Y. Abbas J. Mahaki B. Chaboksavar F. Kianipour N.. (2021). Internet addiction status and related factors among medical students: a cross-sectional study in Western Iran. Int. Q. Commun. Health Educ. 2021, 272684X211025438. 10.1177/0272684X211025438 34128427

[B70] Klasche B.. (2021). After COVID-19: What can we learn about wicked problem governance? Soc. Sci. Human. Open 4, 100173. 10.1016/j.ssaho.2021.100173

[B71] Kniffin K. M. Narayanan J. Anseel F. Antonakis J. Ashford S. P. Bakker A. B.. (2021). COVID-19 and the workplace: implications, issues, and insights for future research and action. Am. Psychol. 76, 63–77. 10.1037/AMP0000716 32772537

[B72] Kumar B. Pinky S. D. Nurudden A. M. (2021). Knowledge, attitudes and practices towards COVID-19 guidelines among students in Bangladesh. Soc. Sci. Human. Open 4, 100194. 10.1016/j.ssaho.2021.100194 34308336 PMC8285241

[B73] Lai H. Pitafi A. H. Hasany N. Islam T. (2021). Enhancing employee agility through information technology competency: an empirical study of China. SAGE Open 11, 21582440211006687. 10.1177/21582440211006687

[B74] Latif K. Malik M. Y. Pitafi A. H. Kanwal S. Latif Z. (2020). If you travel, i travel: testing a model of when and how travel-related content exposure on facebook triggers the intention to visit a tourist destination. SAGE Open 10, 2158244020925511. 10.1177/2158244020925511

[B75] Latif K. Weng Q. Pitafi A. H. Ali A. Siddiqui A. W. Malik M. Y.. (2021). Social comparison as a double-edged sword on social media: the role of envy type and online social identity. Telematics and Informatics 56, 101470. 10.1016/j.tele.2020.101470

[B76] Latif K. F. Nazeer A. Shahzad F. Ullah M. Imranullah M. Sahibzada U. F. (2020). Impact of entrepreneurial leadership on project success: mediating role of knowledge management processes. Leadersh. Organ. Dev. J. 41, 237–256. 10.1108/LODJ-07-2019-0323

[B77] Lebni J. Y. Toghroli R. Abbas J. Kianipour N. NeJhaddadgar N. Salahshoor M. R.. (2021). Nurses' work-related quality of life and its influencing demographic factors at a public hospital in Western Iran: a cross-sectional study. Int. Q. Community Health Educ. 42, 37–45. 10.1177/0272684X20972838 33201756

[B78] Lee J.. (2020). Mental health effects of school closures during COVID-19. Lancet Child Adolesc. Health 4, 421. 10.1016/S2352-4642(20)30109-7 32302537 PMC7156240

[B79] Lee M. Kang B. You M. (2021). Knowledge, attitudes, and practices (KAP) toward COVID-19 : a cross-sectional study in South Korea. BMC Public Health 21, 1–10. 10.1186/s12889-021-10285-y 33546644 PMC7863060

[B80] Li B. Zhong Y. Zhang T. Hua N. (2021). Transcending the COVID-19 crisis: Business resilience and innovation of the restaurant industry in China. J. Hospital Tourism Manag. 49, 44–53. 10.1016/j.jhtm.2021.08.024

[B81] Li H. Cao Y. (2021). Facing the pandemic in the dark: psychopathic personality traits and life history strategies during COVID-19 lockdown period in different areas of China. Curr. Psychol. 1, 1–9. 10.1007/S12144-021-01549-2 33679114 PMC7917003

[B82] Li Z. Wang D. Abbas J. Hassan S. Mubeen R. (2022). Tourists' health risk threats amid COVID-19 era: role of technology innovation, transformation, and recovery implications for sustainable tourism. Front. Psychol. 12, 769175. 10.3389/fpsyg.2021.769175 35465147 PMC9022775

[B83] Liu Q. Qu X. Wang D. Abbas J. Mubeen R. (2022). Product market competition and firm performance: business survival through innovation and entrepreneurial orientation amid COVID-19 financial crisis. Front. Psychol. 12, 790923. 10.3389/fpsyg.2021.790923 35411208 PMC8993680

[B84] Lotfi M. Yousefi A. Jafari S. (2018). The effect of emerging green market on green entrepreneurship and sustainable development in knowledge-based companies. Sustainability 10, 2308. 10.3390/su10072308

[B85] Mahi M. Mobin M. A. Habib M. Akter S. (2021). A bibliometric analysis of pandemic and epidemic studies in economics: future agenda for COVID-19 research. Soc. Sci. Human. Open 4, 100165. 10.1016/j.ssaho.2021.100165 34927059 PMC8665228

[B86] Mamirkulova G. Mi J. Abbas J. (2022). Economic corridor and tourism sustainability amid unpredictable COVID-19 challenges: assessing community well-being in the world heritage sites. Front. Psychol. 12, 797568. 10.3389/fpsyg.2022.797568

[B87] Maqsood A. Abbas J. Rehman G. Mubeen R. (2021). The paradigm shift for educational system continuance in the advent of COVID-19 pandemic: mental health challenges and reflections. Curr. Res. Behav. Sci. 2, 100011. 10.1016/j.crbeha.2020.100011 PMC7832654 38620741

[B88] Marchena-Giráldez C. Acebes-Sánchez J. Román F. J. Granado-Peinado M. (2021). Validation of the spanish version of thework group emotional intelligence profile short version (WEIP-S) in the sports context. Int. J. Environ. Res. Public Health 18, 1–13. 10.3390/IJERPH18020715 33467591 PMC7830954

[B89] Mason A. N. Brown M. Mason K. Narcum J. (2021a). Pandemic effects on social media marketing behaviors in India. Cogent Bus. Manag. 8, 1943243. 10.1080/23311975.2021.1943243

[B90] Mason A. N. Narcum J. Mason K. (2021b). Social media marketing gains importance after Covid-19. Cogent Bus. Manag. 8, 1870797. 10.1080/23311975.2020.1870797

[B91] Mejía-Trejo J.. (2021). COVID-19 ads on purchase intention of online consumer behavior as business innovation activity: A contribution to the uses and gratification theory. Elect. Comm. Res. Appl. 49:101086. 10.1016/j.elerap.2021.101086

[B92] Mirayani R. Kusumaningsih S. W. Mustikasiwi A. Purwanto A. (2019). Transformational, authentic, and authoritarian types of leadership: which one is the most influential in staffs' performance (A Study On Performance In A Religious School Setting). Dinasti Int. J. Educ. Manag. Soc. Sci. 1, 172–182. 10.31933/DIJEMSS.V1I2.68

[B93] Moradi F. Tourani S. Ziapour A. Abbas J. Hematti M. Moghadam E. J.. (2021). Emotional intelligence and quality of life in elderly diabetic patients. Int. Q. Commun. Health Educ. 42, 15–20. 10.1177/0272684X20965811 33086936

[B94] Mubeen R. Han D. Abbas J. Alvarez-Otero S. Sial M. S. (2021a). The relationship between CEO duality and business firms' performance: the moderating role of firm size and corporate social responsibility. Front. Psychol. 12, 669715. 10.3389/fpsyg.2021.669715 35035363 PMC8757377

[B95] Mubeen R. Han D. Abbas J. Hussain I. (2020). The effects of market competition, capital structure, and CEO duality on firm performance: a mediation analysis by incorporating the GMM model technique. Sustainability 12, 3480. 10.3390/su12083480

[B96] Mubeen R. Han D. Abbas J. Raza S. (2021b). Examining the relationship between product market competition and Chinese firms performance: the mediating impact of capital structure and moderating influence of firm size. Front. Psychol. 12, 709678. 10.3389/fpsyg.2021.709678 PMC9156956 35662855

[B97] NeJhaddadgar N. Ziapour A. Abbas J. Mardi A. Zare M. (2020). Correlation between general health and sexual function in older women in an Iranian setting. J Educ Health Promot. 9:300. 10.4103/jehp.jehp_316_20 33426104 PMC7774623

[B98] NeJhaddadgar N. Ziapour A. Zakkipour G. Abbas J. Abolfathi M. Shabani M. (2022). Effectiveness of telephone-based screening and triage during COVID-19 outbreak in the promoted primary healthcare system: a case study in Ardabil province, Iran. Am. J. Public Health. 30:1301–1306. 10.1007/s10389-020-01407-8 33224715 PMC7665795

[B99] Pandya A. Lodha P. (2021). Mental health of college students amid COVID-19: implications for reopening of colleges and universities. Indian J. Psychol. Med. 43, 274–275. 10.1177/02537176211005622 34345109 PMC8287389

[B100] Paulson K. R. Kamath A. M. Alam T. Bienhoff K. Abady G. G. Abbas J.. (2021). Global, regional, and national progress towards Sustainable Development Goal 3.2 for neonatal and child health: all-cause and cause-specific mortality findings from the Global Burden of Disease Study 2019. Lancet 398, 870–905. 10.1016/s0140-6736(21)01207-1 34416195 PMC8429803

[B101] Pitafi A. H. Kanwal S. Akhtar S. Irfan M. (2018a). Investigating the employee work performance in task interdependence and ESM environment. Int. J. Inf. Syst. Change Manag. 10, 266–292. 10.1504/ijiscm.2018.096787

[B102] Pitafi A. H. Kanwal S. Ali A. Khan A. N. Waqas Ameen M. (2018b). Moderating roles of IT competency and work cooperation on employee work performance in an ESM environment. Technol. Soc. 55, 199–208. 10.1016/j.techsoc.2018.08.002

[B103] Pitafi A. H. Kanwal S. Khan A. N. (2020a). Effects of perceived ease of use on SNSs-addiction through psychological dependence, habit: the moderating role of perceived usefulness. Int. J. Bus. Inform. Syst. 33, 383–407. 10.1504/ijbis.2020.105831

[B104] Pitafi A. H. Kanwal S. Pitafi A. (2019). Effect of enterprise social media and psychological safety on employee's agility: mediating role of communication quality. Int. J. Agile Syst. Manag. 12, 1–26. 10.1504/ijasm.2019.098708

[B105] Pitafi A. H. Rasheed M. I. Kanwal S. Ren M. (2020b). Employee agility and enterprise social media: the Role of IT proficiency and work expertise. Technol. Soc. 63, 101333. 10.1016/j.techsoc.2020.101333

[B106] Pitafi A. H. Ren M. (2021). Predicting the factors of employee agility using enterprise social media: moderating effects of enterprise social media-related strain. Intern. Res. 31, 1963–1990. 10.1108/intr-11-2019-0469

[B107] Prempeh C.. (2021). Religion and the state in an episodic moment of COVID-19 in Ghana. Soc. Sci. Human. Open 4, 100141. 10.1016/j.ssaho.2021.100141 34746751 PMC8558695

[B108] Purnama S. G. Susanna D. (2020). Attitude to COVID-19 prevention with large-scale social restrictions (PSBB) in Indonesia: partial least squares structural equation modeling. Front. Public Health 8, 570394. 10.3389/fpubh.2020.570394 33194970 PMC7661637

[B109] Rahmat T. E. Raza S. Zahid H. Abbas J. Mohd Sobri F. Sidiki S. (2022). Nexus between integrating technology readiness 2.0 index and students' e-library services adoption amid the COVID-19 challenges: implications based on the theory of planned behavior. J. Educ. Health Promot. 11, 50. 10.4103/jehp.jehp_508_21 35372596 PMC8974977

[B110] Rasheed M. I. Malik M. J. Pitafi A. H. Iqbal J. Anser M. K. Abbas M. (2020). Usage of social media, student engagement, and creativity: the role of knowledge sharing behavior and cyberbullying. Comput. Educ. 159, 104002. 10.1016/j.compedu.2020.104002

[B111] Rashid R. M. Rashid Q. u. A Pitafi A. H. (2020). Examining the role of social factors and mooring effects as moderators on consumers' shopping intentions in social commerce environments. SAGE Open 10, 2158244020952073. 10.1177/2158244020952073

[B112] Reuter C. Ludwig T. Kaufhold M. A. Spielhofer T. (2016). Emergency services? attitudes towards social media: a quantitative and qualitative survey across Europe. Int. J. Human Comput. Stud. 95, 96–111. 10.1016/J.IJHCS.2016.03.005

[B113] Rizwan M. Ahmad T. Qi X. Murad M. A. Baig M. Sagga A. K.. (2021). Social media use, psychological distress and knowledge, attitude, and practices regarding the COVID-19 among a sample of the population of Pakistan. Front. Med. 8, 1803. 10.3389/FMED.2021.754121/BIBTEX 34746190 PMC8564361

[B114] Rosenberg H. Ophir Y. Asterhan C. S. C. (2018). A virtual safe zone: Teachers supporting teenage student resilience through social media in times of war ^*^. Teach. Teacher Educ. 73, 35–42. 10.1016/j.tate.2018.03.011

[B115] Roy S. Dutta R. Ghosh P. (2021). Identifying key indicators of job loss trends during COVID-19 and beyond. Soc. Sci. Human. Open 4, 100163. 10.1016/j.ssaho.2021.100163 33997770 PMC8112322

[B116] Saha A. Dutta A. Sifat R. I. (2021). The mental impact of digital divide due to COVID-19 pandemic induced emergency online learning at undergraduate level: Evidence from undergraduate students from Dhaka City. J. Affect. Disord. 294, 170–179. 10.1016/J.JAD.2021.07.045 34298222 PMC8433598

[B117] Sarfraz M. Mohsin M. Naseem S. Kumar A. (2021). Modeling the relationship between carbon emissions and environmental sustainability during COVID-19: a new evidence from asymmetric ARDL cointegration approach. Environ. Dev. Sustain. 23, 16208–16226. 10.1007/S10668-021-01324-0 33782633 PMC7989717

[B118] Serafini G. Parmigiani B. Amerio A. Aguglia A. Sher L. Amore M. (2020). The psychological impact of COVID-19 on the mental health in the general population. QJM 113, 229–235. 10.1093/qjmed/hcaa201 32569360 PMC7337855

[B119] Shafi M. Liu J. Ren W. (2020). Impact of COVID-19 pandemic on micro, small, and medium-sized enterprises operating in Pakistan. Res. Global. 2, 100018. 10.1016/J.RESGLO.2020.100018

[B120] Shoib S. Gaitan Buitrago J. E. T. Shuja K. H. Aqeel M. de Filippis R. Abbas J.. (2021). Suicidal behavior sociocultural factors in developing countries during COVID-19. L'Encéphale 47, 78–82. 10.1016/j.encep.2021.06.011 34654566 PMC8457957

[B121] Shuja K. H. Aqeel M. Jaffar A. Ahmed A. (2020). COVID-19 pandemic and impending global mental health implications. Psychiatr. Danub. 32, 32–35. 10.24869/psyd.2020.32 32303027

[B122] Smith J. Guimond F. A. Bergeron J. St-Amand J. Fitzpatrick C. Gagnon M. (2021). Changes in students' achievement motivation in the context of the COVID-19 pandemic: a function of extraversion/introversion? Educ. Sci. 11, 1–8. 10.3390/educsci11010030

[B123] Su Z. McDonnell D. Cheshmehzangi A. Abbas J. Li X. Cai Y. (2021a). The promise and perils of Unit 731 data to advance COVID-19 research. BMJ Glob. Health 6, e004772. 10.1136/bmjgh-2020-004772 34016575 PMC8141376

[B124] Su Z. McDonnell D. Li X. Bennett B. Segalo S. Abbas J.. (2021b). COVID-19 vaccine donations-vaccine empathy or vaccine diplomacy? A narrative literature review. Vaccines. 9, 1024. 10.3390/vaccines9091024 34579261 PMC8470866

[B125] Su Z. McDonnell D. Wen J. Kozak M. Abbas J. Segalo S.. (2021c). Mental health consequences of COVID-19 media coverage: the need for effective crisis communication practices. Global. Health 17, 4. 10.1186/s12992-020-00654-4 33402169 PMC7784222

[B126] Tonkin A. Whitaker J. (2021). Play and playfulness for health and wellbeing: a panacea for mitigating the impact of coronavirus (COVID 19). Soc. Sci. Human. Open 4, 100142. 10.1016/j.ssaho.2021.100142 34927056 PMC8665352

[B127] Wang C. Wang D. Abbas J. Duan K. Mubeen R. (2021). Global financial crisis, smart lockdown strategies, and the COVID-19 spillover impacts: a global perspective implications from southeast asia. Front Psychiatry 12, 643783. 10.3389/fpsyt.2021.643783 34539457 PMC8446391

[B128] Wang Y. Tian T. Pan D. Zhang J. Xie W. Wang S.. (2021). The relationship between dietary patterns and overweight and obesity among adult in Jiangsu Province of China: a structural equation model. BMC Public Health 21, 11341. 10.1186/s12889-021-11341-3 34172040 PMC8229268

[B129] Wei C. Pitafi A. H. Kanwal S. Ali A. Ren M. (2020). Improving employee agility using enterprise social media and digital fluency: moderated mediation model. IEEE Access 8, 68799–68810. 10.1109/access.2020.2983480

[B130] Wilczewski M. Gorbaniuk O. Giuri P. (2021). The psychological and academic effects of studying from the home and host country during the COVID-19 pandemic. Front. Psychol. 12, 644096. 10.3389/fpsyg.2021.644096 33897547 PMC8062758

[B131] World Health Organization (2021). COVID-19 Vaccines. Available online at: https://www.who.int/emergencies/diseases/novel-coronavirus-2019/covid-19-vaccines (accessed January 15, 2022).

[B132] Wu S. Pitafi A. H. Pitafi S. Ren M. (2021). Investigating the consequences of the socio-instrumental use of enterprise social media on employee work efficiency: a work-stress environment. Front. Psychol. 12, 738118. 10.3389/fpsyg.2021.738118 34512489 PMC8428237

[B133] Xiong P. Ming W. K. Zhang C. Bai J. Luo C. Cao W.. (2021). Factors influencing mental health among chinese medical and non-medical students in the early stage of the COVID-19 Pandemic. Front. Public Health 9, 603331. 10.3389/fpubh.2021.603331 34095044 PMC8172592

[B134] Yao J. Ziapour A. Abbas J. Toraji R. NeJhaddadgar N. (2022). Assessing puberty-related health needs among 10?15-year-old boys: A cross-sectional study approach. Arch Pediatr. 10.1016/j.arcped.2021.11.018. [Epub ahead of print]. 35292195

[B135] Ye Z. Yang X. Zeng C. Wang Y. Shen Z. Li X.. (2020). Resilience, social support, and coping as mediators between COVID-19-related stressful experiences and acute stress disorder among college students in China. Appl. Psychol. Health Well-Being 12, 1074–1094. 10.1111/aphw.12211 32666713 PMC7405224

[B136] Yoosefi Lebni J. Abbas J. Moradi F. Salahshoor M. R. Chaboksavar F. Irandoost S. F.. (2021). How the COVID-19 pandemic effected economic, social, political, and cultural factors: a lesson from Iran. Int. J. Soc. Psychiatry 67, 298–300. 10.1177/0020764020939984 32615838 PMC8107447

[B137] Younis A. Xiaobao P. Nadeem M. A. Kanwal S. Pitafi A. H. Qiong G.. (2020). Impact of positivity and empathy on social entrepreneurial intention: the moderating role of perceived social support. J. Public Affairs 21, e2124. 10.1002/pa.2124

[B138] Yousaf M. A. Noreen M. Saleem T. Yousaf I. (2020). A cross-sectional survey of knowledge, attitude, and practices (KAP) toward pandemic COVID-19 among the general population of Jammu and Kashmir, India. Soc. Work Public Health 35, 569–578. 10.1080/19371918.2020.1806983 32970548

[B139] Zhao N. Zhou G. (2021). COVID-19 stress and addictive social media use (SMU): mediating role of active use and social media flow. Front. Psychiatry 12, 85. 10.3389/FPSYT.2021.635546/BIBTEX 33633616 PMC7899994

[B140] Zhong B. L. Luo W. Li H. M. Zhang Q. Q. Liu X. G. Li W. T.. (2020). Knowledge, attitudes, and practices towards COVID-19 among chinese residents during the rapid rise period of the COVID-19 outbreak: a quick online cross-sectional survey. Int. J. Biol. Sci. 16, 1745–1752. 10.7150/IJBS.45221 32226294 PMC7098034

[B141] Zhou Y. Draghici A. Abbas J. Mubeen R. Boatca M. E. Salam M. A. (2021). Social media efficacy in crisis management: effectiveness of non-pharmaceutical interventions to manage COVID-19 challenges. Front. Psychiatry 12, 626134. 10.3389/fpsyt.2021.626134 35197870 PMC8859332

